# A case of gallstones in an African green monkey (*Chlorocebus aethiops*)

**DOI:** 10.5194/pb-4-33-2017

**Published:** 2017-03-08

**Authors:** Dina Kleinlützum, Roland Plesker

**Affiliations:** Paul-Ehrlich-Institut, Paul-Ehrlich-Strasse 51–59, 63225 Langen, Germany

## Abstract

Spontaneous cholelithiasis was found in a male African green monkey (*Chlorocebus aethiops*) at necropsy. Choleliths varied in size, shape and colour. Gallstones were analysed using accepted analytical methods. Results showed that the gallstones were composed of cholesterol and protein in varying proportions. Histologically, the gallbladder showed diffuse mild to moderate lymphocytic infiltration. The etiology of the cholelithiasis in the examined individual remains unknown.

## 1 Introduction

In humans, 10–20 % of the adult population in developed countries harbour gallstones, but less than 20 % are symptomatic (Kumar et al., 2015). Gallstones result from nucleation of biliary solutes (Swidsinski and Lee, 2001; Idris et al., 2014). The exact composition of gallstones depends on the local milieu such as cholesterol saturation and the ratio of cholesterol to bile acids and phospholipids (Chowdhury and Lobo, 2011) and is additionally strongly affected by epidemiological and genetic factors such as gender, nutrition, age, lipid metabolism and anatomical anomalies (Shaffer, 2005, 2006; Tazuma, 2006). Based on these local conditions, gallstones are either classified as cholesterol, pigment or mixed gallstones (Chowdhury and Lobo, 2011). Cholesterol gallstones, accounting for 80 % of gallstones in the western hemisphere, may arise when cholesterol rates exceed the solubilising capacity of bile. Gallbladder stasis and mucus hypersecretion can promote their formation and growth (Wang et al., 2008; Kumar et al., 2015). Furthermore, predisposition to cholesterol gallstones has been ascribed to mutations in various genes including those encoding for enzymes essential for bile acid synthesis (CYP7A1; Pullinger et al., 2002) and genes encoding for ATP-binding cassette transport proteins (ATP transporters), mediating the hepatobiliary secretion of phospholipids (ABCB4; Rosmorduc et al., 2003), bile salts (ABCB11; van Mil et al., 2004) and cholesterol (ABCG5/G8; Buch et al., 2007; Kampen et al., 2013). Pigment stones, containing bilirubin calcium salts, are further divided into two subgroups. Black pigment stones are typically seen in patients with decreased bilirubin conjugation (e.g. cirrhosis, cystic fibrosis) or chronic hemolysis. Brown pigment stones, on the other hand, are linked to biliary infection and stasis and mostly arise in bile ducts. The former type generally contains more cholesterol than black pigment stones (Tazuma, 2006; Chowdhury and Lobo, 2011). In addition, bile infection can alter the gallstone composition towards protein to form a mixed gallstone type (Swidsinski and Lee, 2001).

Spontaneous gallstone formation has been described in Asian and African primates, such as African green monkeys (AGMs), marmosets, baboons, slender lorises, macaques, orangutans and owl monkeys (Baer et al., 1990; Plesker et al., 2012). The Brazilian squirrel monkey, which is highly susceptible to spontaneous gallstone formation when fed atherogenic diets, has been used successfully as a model in cholelithiasis studies (Osuga and Portman, 1971; Tanaka et al., 1976). Meanwhile, these historical model animals have been replaced by rodent models such as the Syrian hamster and mouse models (Combettes-Souverain et al., 2002; Wang and Lee, 2008). However, cholelithiasis and its associated disorders appear to be less common in primates compared to humans (Smith et al., 2006). To date, diet-induced cholesterol gallstones have been described in AGMs (Rudel et al., 1994, 2002), whereas little is known about spontaneous gallstones in AGMs, especially pigment and mixed gallstones. Here, we report a naturally occurring case of cholelithiasis in a male AGM.

## 2 Methods and materials

### 2.1 Case history

A 20-year-old male AGM, born in the Central Animal Facility of the Paul-Ehrlich-Institut, was infected with simian immunodeficiency virus (SIV) at the age of 30 months. The animal was kept alone due to aggression towards other monkeys. Housing, handling and experimental procedures were performed in accordance with European regulations.

The animal was humanly euthanised according to standard protocols. Before death, the animal weighed 4.5 kg. Body temperature was 39.7 ${}^{\circ }$C (reference source 37.5–39.3). No specific clinical signs indicating liver–gallbladder problems were identified before death.

### 2.2 Feeding

Briefly, the feeding procedure consisted of ad libitum offering of pellets (ssniff, Primaten vegetarisch 10 mm; ssniff Spezialdiäten GmbH, Soest, Germany) in the morning between 07:00 and 08:00. Twice per week, the monkey was offered fruits and vegetables. At 14:00, a handful of a grain mixture (55 % wheat, 5 % barley, 27 % maize, 3 % sunflower kernels, 3 % peas, 1 % rapeseed, 3 % oat and 2 % shrimp) were thrown into the bedding of the cage for enrichment purposes.

### 2.3 Necropsy and histology

The necropsy was performed immediately after the death of the animal. Organ samples were routinely fixed in a 4 % formaldehyde solution, embedded in paraffin and sectioned. Tissue sections (thickness 4 µm) were stained with hematoxylin and eosin as well as Masson trichrome using standard methods.

### 2.4 Chemical analyses of the gallstones

Analyses of gallstones were performed in a commercial laboratory (IDEXX Vet Med Labor, Ludwigsburg, Germany), which used the Fourier transform infrared (FT-IR) spectrometer IS5 coupled with attenuated total reflection (ATR).

**Figure 1:**
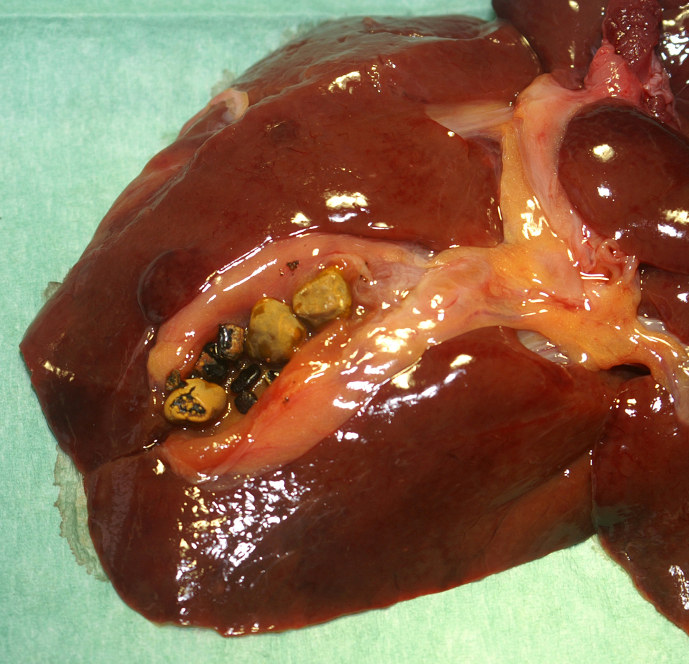
Gallbladder of an adult male African green monkey with multiple light brown gallstones with a black core and several black irregular fragments.

### 2.5 Microbiology

The gallstones were stored for 6 weeks prior to culturing (Institute of Hygiene and Infectious Diseases of Animals, Justus Liebig University Giessen, Germany). Briefly, the gallstones were manually disrupted with a sterile mortar and pestle. For the aerobic culture the obtained material was streaked on a blood agar plate containing 5 % defibrinated sheep blood and on a Gassner agar plate (both Merck, Darmstadt, Germany). For the anaerobic culture material was streaked on a Schaedler agar plate with 5 % sheep blood (Beckon and Dickinson, Heidelberg, Germany) and on a blood agar plate containing 1 % dextrose (according to Zeissler). Agar plates were incubated as recommended by the manufacturer. Additionally, enrichment of the sample material was conducted using a Rappaport-Vassiliadis broth (Oxoid, Wesel, Germany) and tetrathionate broth according to Müller-Kaufmann (Merck, Darmstadt, Germany), followed by *Salmonella* spp. culture according to standard protocols.

## 3 Results

### 3.1 Necropsy and histology

At necropsy, no concomitant organ pathologies associated with gallbladder pathology, e.g. jaundice or ascites, were noted. Incision of the gallbladder revealed three light brown gallstones measuring 5 × 5 × 3 mm each. These appeared to have a black core and were partially solid at varying stages of disintegration. In addition, several irregular black pinpoint to pinhead sized fragments were noted (Fig. 1). Although the gallbladder was filled, no blockage of the bile duct could be confirmed macroscopically.

At histological examination, the gallbladder showed a moderate chronic diffuse hyperplasia of the gallbladder epithelium. Also, a moderate diffuse infiltration of lymphocytes was noted. A Masson trichrome stain revealed a slight fibrosis of the subepithelial stroma. The liver showed slight pericholangiolar fibrosis of the subepithelial stroma and a few multifocal lymphocytic infiltrates.

### 3.2 Chemical analyses of the gallstones

The chemical composition of the gallstones differed significantly between the three large and the multiple small irregular stones. The chemical analysis revealed differences between the core and the sheath: the envelope of the light brown gallstones was composed of more than 30 % cholesterol, whereas the core contained more than 50 % cholesterol. However, the small irregular gallstones showed no difference in their composition between core and sheath and contained less than 20 % cholesterol. The remaining mass of the gallstones consisted of protein.

**Figure 2:**
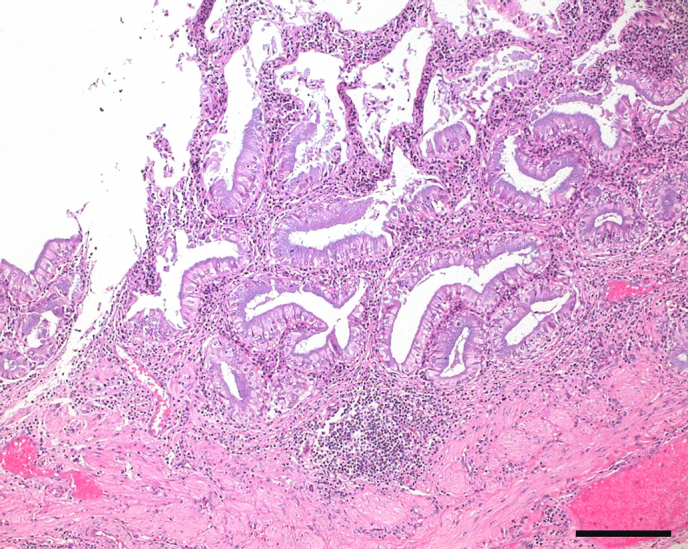
Gallbladder histology. Section showing chronic lymphocytic cholecystitis as characterised by hyperplastic epithelium and mild fibrosis. Hematoxylin and eosin stain; scale bar = 200 µm.

### 3.3 Microbiology

The aerobic and anaerobic cultures as well as the screen for *Salmonella* spp. were negative.

## 4 Discussion

The pathogenesis of gallstones is not fully understood, but gallstones develop due to an imbalance of biliary components, leading to supersaturation and subsequent precipitation. However, lithogenesis is complex and seemingly involves not only malnutrition and other dietary factors, but also bile infection (Tazuma, 2006). Gallstones may also form as a result of biliary stasis due to hypomotility, parasitic or bacterial infection or bile duct obstruction. Bacteria may contribute to gallstone pathogenesis through the degradation of bilirubin glucuronide (Swidsinski, 1992; Swidsinski and Lee, 2001; Swidsinski et al., 1995). In approximately 90 % of cases, cholecystitis occurs (Prevot, 2014). Moreover, gallstones are known to be an important risk factor for gallbladder cancer (Misra et al., 2003).

As in humans, cholelithiasis is the most prevalent gallbladder pathology in monkeys (Slingluff et al., 2010). Indeed, spontaneous cholelithiasis has been reported in several species of primates (Anver et al., 1972; Baer et al., 1990; Pissinatti et al., 1992; Smith et al., 2006; Slingluff et al., 2010; Plesker et al., 2012; Lieberman et al., 2016). Although little is known about gallstones in AGMs, in some aspects the development of gallstones in humans and in susceptible species such as some Saimiri species (Lieberman et al., 2016) and AGMs share similarities. As in most human cases, females are disproportionately affected and gallstone formation is more frequent in elderly animals (Tazuma, 2006).

In contrast to the most frequent epidemiological correlations, we describe a case of spontaneous gallstones in a 20-year-old male AGM that was otherwise asymptomatic. It is probable that pain associated with gallstone disease in monkeys may be unrecognised since monkeys are known to mask pain in order to appear fit within a group (Plesker and Mayer, 2008). The lack of a high-cholesterol diet and cholesterol proportions below 80 % suggest pigment or mixed gallstone genesis (Idris et al., 2014). Moreover, the animal was not fed lithogenically. Mixed and pigment gallstones are known to be attributed mainly to biliary stasis caused by infection (Tazuma, 2006). In our case, the only sign of an inflammatory event was the lymhocytic cholecystitis, the hyperplastic gallbladder epithelium and the hepatic lymphocytic infiltrates, which are indicative of a viral etiology rather than a bacterial or parasitic cause. The lack of viral inclusion bodies on liver and gallbladder histopathology make a viral etiology unlikely in this case. Although our animal was seropositive for simian immunodefiency virus (SIV), an SIV etiology is unlikely. Many African green monkeys in our colony are positive for SIV. However, this is the only individual in which gallstones were detected, which indicates that there may be no correlation between SIV infection and gallstone development. A bacterial cause for initial gallstone formation cannot be ruled out, but no evidence of pus or purulent bile was found and the microbiological culture was negative. It is often not possible to ascertain whether an infection of bile initiates gallstone formation or vice versa, but gallstones are likely to perpetuate a “vicious cycle”-style inflammation. It has been suggested that the chemical composition of choleliths may not be fixed but are rather alterable throughout the gallstone harbour (Swidsinski, 1992). According to the multi-step proposal, gallstone formation (nucleation, assembly of microcalculi, growth, remodelling) includes the interaction of both bacterial and non-bacterial mechanisms. Lithogenesis may therefore be initiated by an infectious process and may develop further either into mixed or cholesterol gallstones depending on the predominantly depositing type of concrement. Likewise, they may also act as a surface for bacterial colonization and biofilm development, thereby providing a reservoir for infection (Crawford et al., 2010; Gonzalez-Escobedo and Gunn, 2013; Swidsinski, 1992; Swidsinski and Lee, 2001) despite negative culture findings.

In the case presented here, we presume that the small gallstones found are disintegration products of the larger gallstones. Altogether, we found two main size ranges: a large size range (5 × 5 × 3 mm), consisting of more than 50 % cholesterol, and a small size range (pinhead to pin point), consisting of less than 20 % cholesterol. The component ratios between large and small stones, as well as between core and sheath, indicate a batch formation of concrements as described above (Swidsinski and Lee, 2001). However, due to the lack of further investigations into elements, such as protein detection of choleliths, liver chemistry and bacterial culture of bile, we cannot confirm the definite gallstone type and the underlying etiology. Except for the mild epithelial hyperplasia, we did not observe indications of epithelial dyplasia, atypical hyperplasia or malignant transformation of the gallbladder tissue. We therefore assume that the gallstone disease in the case described here would not have been fatal.

## 5 Data availability

The diagnostic reports of the chemical analyses of the gallstones as well as of the microbiology used in this study are available in the Supplement.

## Supplement

10.5194/pb-4-33-2017-supplement The supplement related to this article is available online at: https://doi.org/10.5194/pb-4-33-2017-supplement.
