# Supplementary material for: A case of gallstones in an African green monkey (Chlorocebus aethiops)
Source: Primate Biol. 2017 Mar 8;4(1):33–7. doi: 10.5194/pb-4-33-2017 (PMC7041542; doi:10.5194/pb-4-33-2017)
Supplement: The supplement related to this article is available online at: https://doi.org/10.5194/pb-4-33-2017-supplement. [file pb-4-33-supplement.zip › Chemical analysis of gallstones.pdf]

Paul-Ehrlich-Institut
Paul-Ehrlich-Str. 51-59
63225 Langen

Tierhalter	**Paul- Ehlich- Institut**	Auftrags-Nr	
Tier	**Affe**	Labor-Nr / Probeneingangsdatum	**VM790387/15.04.2016**
Material	**Stein**		

Untersuchung	Ergebnis	Sign	Normalwert	Maßeinheit	Anm
**Steinanalyse (Infrarotspektroskopie)**					
Anzahl	**2**				
Größe	**6x7**			mm	
Farbe	**braun**				
Oberfläche	**rauh**				
Konsistenz	**weich**				
**Chemische Zusammensetzung (Infrarotspektroskopie)**					
Hauptbestandteil	**siehe unten**				
grosse Stein:<br>Innen dunkelbraun => Cholesterol > 50% Protein <50%<br>Aussen hellbraun => Cholesterol > 30 % Protein >60 % nicht identifiziertbar <1<br>0 %<br>kleiner Stein:<br>kein Unterschied zwischen Innen und Aussen<br>dunkelbraun => Protein >80%;Cholesterol <20 %					
Anteil	**siehe unten**			%	

***** Endbefund ***** validiert von Dr. Keidel **(Tierärztin)**
Dieser Befund wurde elektronisch erstellt und ist auch ohne Unterschrift gültig.
Angaben zum genauen Untersuchungszeitpunkt können jederzeit erfragt werden.
Alle nicht gesondert gekennzeichneten Untersuchungen wurden am Standort Ludwigsburg als akkreditierte Untersuchungen durchgeführt.
Achtung, ab sofort können Sie uns kostenlos unter 0800 5892579, Option 1 erreichen!

Vorläufige Kostenaufstellung:		
Steinanalyse (SA)		19,30 EUR
Kurier-Probenabholung		3,50 EUR
	Summe	22,80 EUR
	+ MwSt 19,0 %	4,33 EUR
	Rechnungsbetrag	**27,13 EUR**

Rechnungsstellung erfolgt mit nächster Sammelrechnung.
Rabatte sind hier nicht berücksichtigt.

Jetzt Fahrdienst via Smartphone anfordern!
Einfach http://fahrdienst.idexx.de als Bookmark speichern oder Anleitung über die Hotline anfordern.

Vet Med Labor GmbH
Division of IDEXX Laboratories

Standort	Standort	**tel: 0800/5892579**
Labor Ludwigsburg	Labor Leipzig	**fax: 07141/6483555**
Mörikestr. 28/3	Druckereistr. 4	web: IDEXX Vet Med Labor
71636 Ludwigsburg	04159 Leipzig	email: IDEXX Vet Med Labor

Generated by C/lab mfs2html p0783ps.vm on 15.04.16 17:15:53
