# Supplementary material for: A case of gallstones in an African green monkey (Chlorocebus aethiops)
Source: Primate Biol. 2017 Mar 8;4(1):33–7. doi: 10.5194/pb-4-33-2017 (PMC7041542; doi:10.5194/pb-4-33-2017)
Supplement: The supplement related to this article is available online at: https://doi.org/10.5194/pb-4-33-2017-supplement. [file pb-4-33-supplement.zip › Microbiology.pdf]

Prof. Dr. Christa Ewers
Fachtierärztin für Mikrobiologie
Institut für Hygiene und Infektionskrankheiten der Tiere
der Justus-Liebig-Universität Gießen
Frankfurter Str. 85-89
D-35392 G i e ß e n

Fax: 0641/99 383 09
Tel.: Bakt. Labor: 0641/99 383 11
Serol. Labor: 0641/99 383 12/05

Untersuchungsantrag

Einsender: (genaue Anschrift/Stempel !)

Dr. Plesker
Paul-Ehrlich-Institut
Paul-Ehrlich-Str. 51-59
63225 Langen

Patient: (Tierart, Name, Alter)
Afrikanische Grüne Meerkatze

Art des Untersuchungsmaterials:
Gallensteine

Klinische Diagnose / Verdacht auf:
....

Rechnung an: Einsender: ☒
Besitzer: ☐

Neue Vordrucke ☐

Herstellung von Vakzinen: ☐
(z. B. *E. coli*, *Staph. aureus*)

Tgb.-Nr.: P7367
(bei Rückfragen bitte angeben)
22.11.16

Tierbesitzer: (genaue Anschrift !)

Name:

Straße:

PLZ: Ort:

Erwünschte Untersuchung: (bitte ankreuzen)

bakteriologisch .... ☐ **Resistenztest: Ja** ☐ **nein** ☐
speziell auf.............................. ☐
mykologisch ☐

Nachweis von Antikörpern auf/gegen:

Leptospira interrogans ☐
Chlamydia psittaci ☐
Listeria monocytogenes ☐
Borrelia burgdorferi ☐
Coxiella burnetii ☐
Brucella canis ☐
Brucella abortus/melitensis ☐

Paratuberkulose ☐
Rotlauf ☐
Tularämie ☐
.... ☐

Nachweis von spezifischen DNA-Sequenzen (PCR):

☐ *Escherichia coli* ☐ *Brachyspira*/*Lawsonia* sp.
☐ *Chlamydia* sp. ☐ *Coxiella burnetii*
☐ Sonstige: ....

Datum: 22.11.16 Plesker
Unterschrift (Einsender)

Ergebnis der mikrobiologischen Untersuchung:

(Bitte freie Fläche nicht beschreiben!)

☐ Resistenztest siehe Rückseite bzw. Extrablatt

Bakterienwachstum konnte auch nach Serumbouillonanreicherung **nicht** nachgewiesen werden.

Gebühren: EURO 13,-- (+MwSt)
(gilt nicht als Rechnung)

Gießen, den 25.11.16

....
(Prof. Dr. Christa Ewers)
