# Supplementary material for: A case of gallstones in an African green monkey (Chlorocebus aethiops)
Source: Primate Biol. 2017 Mar 8;4(1):33–7. doi: 10.5194/pb-4-33-2017 (PMC7041542; doi:10.5194/pb-4-33-2017)
Supplement: The supplement related to this article is available online at: https://doi.org/10.5194/pb-4-33-2017-supplement. [file pb-4-33-supplement.zip › pb-4-33-2017-supplement-title-page.pdf]

Supplement of Primate Biol., 4, 33–37, 2017
http://www.primate-biol.net/4/33/2017/
doi:10.5194/pb-4-33-2017-supplement
© Author(s) 2017. CC Attribution 3.0 License.

Supplement of

A case of gallstones in an African green monkey (*Chlorocebus aethiops*)

Dina Kleinlützum and Roland Plesker

Correspondence to: Dina Kleinlützum (dina.kleinluetzum@pei.de)

- pb-4-33-2017-supplement-title-page.pdf
- Chemical analysis of gallstones.pdf
- Microbiology.pdf

The copyright of individual parts of the supplement might differ from the CC-BY 3.0 licence.
